# Four hour creatinine clearance is better than plasma creatinine for monitoring renal function in critically ill patients

**DOI:** 10.1186/cc11391

**Published:** 2012-06-19

**Authors:** John W Pickering, Christopher M Frampton, Robert J Walker, Geoffrey M Shaw, Zoltán H Endre

**Affiliations:** 1 Christchurch Kidney Research Group, Department of Medicine, University of Otago, Riccarton Avenue, Christchurch 8140, New Zealand; 2 Department of Medicine and Surgery, University of Otago, Leith Walk, Dunedin 9054, Dunedin, New Zealand; 3 Intensive Care, Christchurch Hospital, Riccarton Avenue, Christchurch 8140, New Zealand; 4 Department of Nephrology, Prince of Wales Clinical School, University of New South Wales, Barker St, Randwick, Sydney, NSW 2031, Australia

## Abstract

**Introduction:**

Acute kidney injury (AKI) diagnosis is based on an increase in plasma creatinine, which is a slowly changing surrogate of decreased glomerular filtration rate. We investigated whether serial creatinine clearance, a direct measure of the glomerular filtration rate, provided more timely and accurate information on renal function than serial plasma creatinine in critically ill patients.

**Methods:**

Serial plasma creatinine and 4-hour creatinine clearance were measured 12-hourly for 24 hours and then daily in 484 patients. AKI was defined either as > 50% increase in plasma creatinine from baseline, or > 33.3% decrease in creatinine clearance. The diagnostic and predictive performance of the two AKI definitions were compared.

**Results:**

Creatinine clearance decrease diagnosed AKI in 24% of those not diagnosed by plasma creatinine increase on entry. These patients entered the ICU sooner after insult than those diagnosed with AKI by plasma creatinine elevation (*P *= 0.0041). Mortality and dialysis requirement increased with the change in creatinine clearance-acute kidney injury severity class (*P *= 0.0021). Amongst patients with plasma creatinine < 1.24 mg/dl on entry, creatinine clearance improved the prediction of AKI considerably (Net Reclassification Improvement 83%, Integrated Discrimination Improvement 0.29). On-entry, creatinine clearance associated with AKI severity and duration (*P *< 0.0001) predicted dialysis need (area under the curve: 0.75) and death (0.61). A > 33.3% decrease in creatinine clearance over the first 12 hours was associated with a 2.0-fold increased relative risk of dialysis or death.

**Conclusions:**

Repeated 4-hour creatinine clearance measurements in critically ill patients allow earlier detection of AKI, as well as progression and recovery compared to plasma creatinine.

**Trial Registration:**

Australian New Zealand Clinical Trials Registry ACTRN012606000032550.

## Introduction

Acute kidney injury (AKI) is common in critically ill patients and is frequently fatal [1-5]. Although defined as an abrupt decrease in glomerular filtration rate (GFR) [6,7] there are no real time measures of GFR to enable timely diagnosis. In practice, diagnosis depends on observing an increase in plasma creatinine (pCr); according to creatinine kinetics, this may not become apparent until 24 to 72 hours after a decrease in GFR [8]. This temporal disconnect between changed GFR and pCr is readily observable, particularly where there is a clearly defined time of injury, such as cardiopulmonary bypass surgery. The relationship is less clearly demonstrated following multiple or continuing injury and after vigorous resuscitation. The relationship between change in GFR and change in plasma creatinine has not been investigated in critically ill patients at high risk of AKI. In contrast, numerous urinary and plasma biomarkers of kidney injury are under investigation, and are usually assessed by their ability to predict an increase in creatinine [9,10]. Although, many biomarkers show promise as predictors of change in renal function, of dialysis need and of mortality, their primary biological role is to mark the presence of renal injury. With the exception of plasma cystatin C, these biomarkers are not markers of function.

Creatinine clearance (CCl) is an easy to estimate GFR in the intensive care unit, since most patients are catheterised and have frequent measurements of pCr. In patients with normal creatinine a low CCl may be an early indicator of AKI [11]. Several studies have shown that short duration (1 to 4 h) CCl measures are feasible in the critically ill [11-14]. Several of these evaluated CCl by comparing short duration clearance with 24-h clearance [12-14]. While validating the brief clearance technique, these studies did not evaluate the use of brief CCl in the detection of AKI.

Evaluation of 4-h CCl was a planned component of the two-centre Early intervention in Acute Renal Failure (EARLYARF) randomised controlled trial of high dose erythropoietin for the prevention of AKI in the ICU [15]. We hypothesised that CCl would give more timely and accurate information on renal function than pCr. We compared these metrics in the diagnosis of AKI and AKI severity and as predictors of the need for dialysis and mortality. We also compared these metrics with urine output. Finally, we compared serial measurements of CCl with serial measurements of pCr.

## Materials and methods

The study was approved by the multiregional ethics committee of New Zealand (MEC/050020029) and registered under the Australian New Zealand Clinical Trials Registry (ACTRN012606000032550 [16]). Screening on entry to the ICU was by presumptive consent, followed by written consent from the patient or family.

Inclusion and exclusion criteria, consent procedures, estimation of time after renal injury, and creatinine assays have been described in detail elsewhere [15]. Briefly, patients were excluded if they were not expected to remain in the ICU for 24 h or to survive 72 h; anuric; receiving renal replacement therapy, or had obvious haematuria, rhabdomyolysis or polycythemia. There was no significant difference between means or standard deviations of creatinine reference samples between the laboratories at the two centres. Since erythropoietin had no effect on outcome in the EARLYARF trial, this analysis includes patients in both observation and intervention arms [15]. Plasma and urine samples were taken for assay and a 4-h urine collection commenced on entry to ICU at 12 and 24 h post-entry and then daily for 7 days. CCl was calculated in ml/min (Table 1). Baseline renal function in patients with known baseline creatinine was determined by the Cockcroft-Gault (CG) equation [17]. The average urine output per kg of body-weight (UO in ml/kg/hr) was measured on entry to the ICU (Table 1).

**Table 1:** Definitions

Name	Abbreviation	Calculation
Creatinine Clearance	CCl	(Urine creatinine concentration/Plasma creatinine concentration) × (Volume of urine collected over 4 hours in ml/(4 × 60))
Cockcroft-Gault clearance [17]	CG	[(140-Age in years) × weight in kg/(72 × Plasma creatinine concentration in mg/dl)] × 0.85 if female
Average urine output	UO	Volume of urine collected over 4 hours in ml/(4 × Patient weight in kg)
Change in CCl	ΔCCl	100 × (Measured CG on entry - baseline CCl)/Baseline CG
Change in plasma creatinine	ΔpCr	100 × (On entry creatinine - baseline creatinine)/Baseline creatinine
AKI plasma creatinine	ΔpCr_AKI_	ΔpCr > 50%
AKI creatinine clearance	ΔCCl_AKI_	ΔCCl < -33.3%
AKI urine output (oliguria)	UO_AKI_	Urine output < 0.5 ml/kg/hr on average over 4 hours
RIFLE class R	R	ΔpCr > 50% and ≤ 100%, or ΔCCl < -33.3% and ≥ -50%
RIFLE class I	I	ΔpCr > 100% and ≤ 200%, or ΔCCl < -50% and ≥ -66.7%
RIFLE class F	F	ΔpCr > 200% or ΔCCl < -66.7%
AKI from entry (AKIN)	AKI_AKIN_	(Plasma creatinine - on entry plasma creatinine) > 0.3 mg/dl or 50% within 48 hours

AKI: acute kidney injury; RIFLE: Risk, Injury, Failure, Loss, End stage.

### Cohorts with known and unknown baseline creatinine

The cohort was divided into those with a known baseline creatinine (*n *= 182) and those without (*n *= 302). Known baseline creatinine was defined as a measured value within one year of entry to the ICU (*n *= 162), or for elective surgery patients, the pre-surgery sample (*n *= 20).

### Definitions of AKI

The known baseline creatinine cohort was used to test the hypothesis that the estimated change in CCl was a better predictor of outcomes than the change in creatinine (ΔpCr). AKI was defined by an increase in pCr (ΔpCr_AKI_), or an estimated decrease in CCl (ΔCCl_AKI_), or as oliguria (UO_AKI_). Each component approximates a diagnostic criterion of the Risk, Injury, Failure, Loss, End stage (RIFLE) definition [6,18]. Because urine output was measured over 4 h only, oliguria was only defined over this duration rather than the 6-h RIFLE period. Patients were further classified into (i) no AKI (ΔpCr_No-AKI _and ΔCCl_No-AKI_); (ii) AKI by the clearance criterion only (ΔpCr_No-AKI _and ΔCCl_AKI_); (iii) AKI by the pCr criterion only (ΔpCr_AKI _and ΔCCl_No-AKI_) or (iv) AKI by both criteria (ΔpCr_AKI _and ΔCCl_AKI_). Finally, patients were classified according to AKI RIFLE severity class (Table 1).

### CCl and pCr cut-points and risk prediction models

The area under the receiver operator characteristic curve (AUC) was used to determine the predictive value of on-entry CCl for ΔCCl_AKI _and on-entry pCr for ΔpCr_AKI_. For each metric the optimal cut-point was determined as the value nearest to a sensitivity and specificity of one. We were most interested in whether the addition of CCl to existing measurements of pCr and UO helps identify patients with AKI when pCr is low. Therefore, in the cohort with pCr less than the optimal cut-point, a reference risk prediction model (logistic regression) for AKI (either ΔCCl_AKI _or ΔpCr_AKI_) was constructed using pCr, UO and other variables associated with AKI on univariate analysis. The model calculates for each patient the probability of having AKI. To assess the added benefit of CCl, a new model was constructed by adding CCl to the reference model and was compared with the reference model.

Using the entire cohort, the on-entry creatinine and on entry CCl and pCr were compared as predictors of AKI and severity stage according to the AKIN (Acute Kidney Injury Network) criteria [7] (AKI_AKIN_), duration of AKI_AKIN_, death within 30 days and 365 days, need for dialysis, and length of ICU stay.

### Statistical analysis

Results are presented as means ± SD or medians and inter-quartile range (IQR), or incidence presented as number (n). Cohorts were compared with the Student's *t*-test (for normally distributed continuous variables), the Mann-Whitney *U*-test (for variables not normally distributed), and the chi square (χ^2^) or Fisher's exact test for categorical variables. Length of ICU stay was log transformed as necessary for assessment of association with AKI severity class, using one-way analysis of variance (ANOVA). Diagnostic and prognostic performance was assessed by calculating the AUC and odds ratios. The reference and new AKI risk prediction models were compared by the continuous (category-free) net reclassification improvement (NRI) and integrated discrimination improvement statistics [19-21] and difference in AUC [22]. GraphPad Prism 5.0a for Mac OS (GraphPad Software, San Diego, CA, USA) and Matlab 2011a (MathWorks, Natick, MA, USA) were used for statistical analyses. All confidence intervals (CIs) are 95%.

## Results

Of the 528 patients enrolled in the EARLYARF trial, 484 had a CCl measure on entry to the ICU. Of the remainder, 30 were a sub-cohort of high-risk patients who had undergone cardio-thoracic surgery, and whose first clearance measure was 8 to 11 h after entry to ICU, and 14 patients had no clearance measurement because they were anuric or because clinical events prevented measurement. The analysis is based on 484 patients. Patient characteristics are shown in Table 2.

**Table 2:** Patient demographics (*n *= 484) on entry to the ICU

Age, yrs	60 ± 17
Female, % (n)	39 (190)
Weight, kg	79 ± 19
Baseline pCr, mg/dl	0.86 (0.71, 1.06)
Baseline estimated CCl, ml/min	89 (66, 125)
APACHE II score	18 ± 6
SOFA score	6.3 ± 2.8
Hypotension, % (n)	23 (111)
pCr, mg/dl	1.0 (79.0, 1.36)
4-h CCl, ml/min	78 (48, 122)
Urine output, ml/kg/hr	1.0 (52, 2.14)
Urine creatinine, mg/dl	62 (30, 107)
Plasma cystatin C, mg/dl	86 (66, 1.2)
CKD, % (n)	14 (66)
Primary diagnosis, % (n)	

Abdominal aortic aneurysm rupture & repair	5 (22)
Abdominal surgery or inflammation	11 (51)
Burns	1 (5)
Cardiac arrest or failure	13 (63)
Cardiac surgery	13 (64)
Collapse, cause unknown	1 (3)
Neurological surgery, injury or seizure or haemorrhage	15 (71)
Other	1 (3)
Pulmonary or thoracic surgery or failure	13 (63)
Sepsis	20 (97)
Trauma*^a^*	9 (42)

Shown are means ± SD or medians (lower quartile, upper quartile) for normal and non- normally distributed data, or percentage (n).

pCr: plasma creatinine; CCl: creatinine clearance; CKD: chronic kidney disease; APACHE: acute physiology and chronic health evaluation SOFA: Sequential Organ Failure Assessment.

### AKI on entry to ICU (known baseline creatinine cohort)

**Figure 1:**
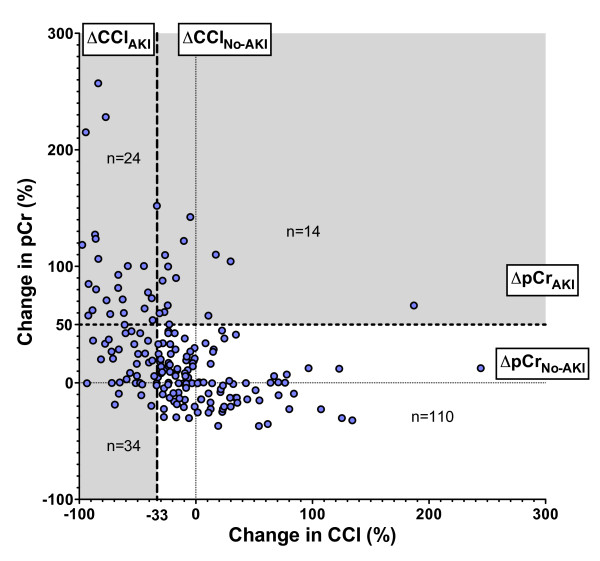
**Comparison of percentage increase in plasma creatinine (pCr) with percentage decrease in creatinine clearance (CCl) from known baseline to entry into the ICU**. Four quadrants are shown: (i) No AKI (ΔpCr_No-AKI _and ΔCCl_No-AKI_), (ii) AKI by the clearance criterion only (ΔpCr_No-AKI _and ΔCCl_AKI_), (iii) AKI by the pCr criterion only (ΔpCr_AKI _and ΔCCl_No-AKI_) or (iv) AKI by both criteria (ΔpCr_AKI _and ΔCCl_AKI_). AKI: acute kidney injury; No-AKI (CCl_No-AKI_): without AKI; ΔpCr: relative change in pCr from baseline; ΔCCl: relative change in CCl from Cockcroft-Gault (CG) baseline; AKI (pCr_AKI_): with AKI.

On entry to the ICU, 182 patients had a pre-admission baseline creatinine from which the change in creatinine (ΔpCr) and change in creatinine clearance (ΔCCl) to determine AKI status on entry was calculated (Figure 1). The ΔpCr only poorly approximated that expected from ΔCCl (r^2 ^= 0.18) according to the creatinine kinetic model [23]:

ΔpCr = 100*[1/(1 + ΔCCl/100) - 1]

**Figure 2:**
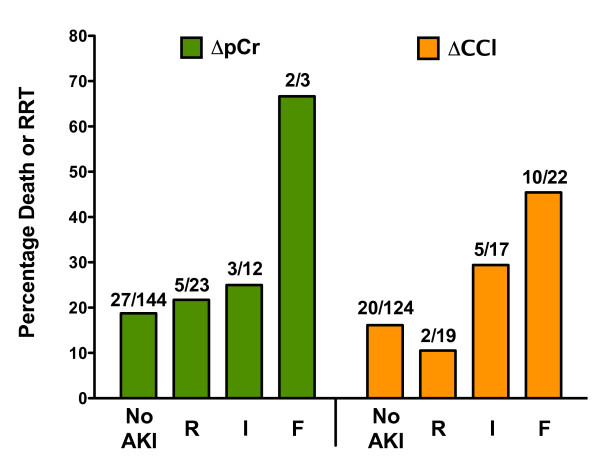
**Comparison of plasma creatinine (pCr) change with estimated creatinine clearance (CCl) change acute kidney injury (AKI) severity classes (No-AKI, R, I or F) on entry to the ICU in the known baseline creatinine cohort**. Percentage of patients who died or needed dialysis; ΔpCr, *P *= 0.12, ΔCCl, *P *= 0.0055 (χ^2 ^test for trend). The number above each bar is the number of patients who died/total number.

Ninety-two patients (51%) had AKI according to ΔCCl_AKI_, ΔpCr_AKI _or UO_AKI_. Thirty-seven percent (*n *= 14) of ΔpCr_AKI _were not simultaneously ΔCCl_AKI_, whereas 24% (*n *= 34) of ΔpCr_No-AKI _had AKI according to ΔCCl_AKI _(Table 3). Twenty more patients were classified as AKI by ΔCCl_AKI _than by ΔpCr_AKI _(McNemar's test *P *< 0.01). Although 14 more patients were classified as UO_AKI _than ΔpCr_AKI_, and a 6 more by ΔCCl_AKI _than UO_AKI_, the differences were not significant (*P *= 0.07 and *P *= 0.49 respectively). Sixteen patients had AKI by all three definitions. Ten of these sixteen patients died or needed dialysis within 30 days (relative risk (RR) 4.1 compared with not meeting all three definitions; 95% CI, 2.7 to 6.4). ΔCCl_AKI _severity classifications were associated with increased 30-day mortality or need for dialysis (χ^2 ^test for trend, *P *= 0.0021), but not ΔpCr_AKI _(*P *= 0.12) (Figure 2). Length of ICU stay was not associated with ΔCCl_AKI _(*P *= 0.49) or ΔpCr_AKI _severity classifications (*P *= 0.95).

**Table 3:** Cross-tabulation of Risk, Injury, Failure, Loss, End stage (RIFLE) on entry in the known baseline creatinine cohort according to the definition

	ΔCCl_No-AKI_	ΔCCl_AKI_	UO_No-AKI_	UO_AKI_
ΔpCr_No-AKI_	110	34	111	33
ΔpCr_AKI_	14	24	19	19
				
ΔCCl_No-AKI_			101	23
ΔCCl_AKI_			29	29

AKI: acute kidney injury; AKI (pCr_AKI_): with AKI; No-AKI (CCl_No-AKI_): without AKI; CCl: creatinine clearance; pCr: plasma creatinine.

The time from insult until entry to the ICU differed between groups (*P *= 0.0041, Kruskal Wallis non-parametric ANOVA). Thirty-four patients were ΔCCl_AKI _and ΔpCr_No-AKI_. Their median time from insult until entry to the ICU, 10.8 h (IQR, 5.0 to 20.0 h), was less than those classified as ΔpCr_AKI _and ΔCCl_AKI_, for whom the equivalent figure was 24.6 h (IQR, 17.0 to 50.0 h) (*P *= 0.0037; Mann-Whitney *U*-test) and was less than ΔpCr_AKI _and ΔCCl_No-AKI_, at 18.7 h (IQR, 9.8 to 48.0 h), but not significantly so (*P *= 0.079). Four ΔCCl_AKI _and ΔpCr_No-AKI _patients developed AKI according to ΔpCr at a later time point, and three further patients died within seven days.

### CCl as a risk factor for AKI on entry to ICU when pCr is low (known baseline creatinine cohort)

**Figure 3:**
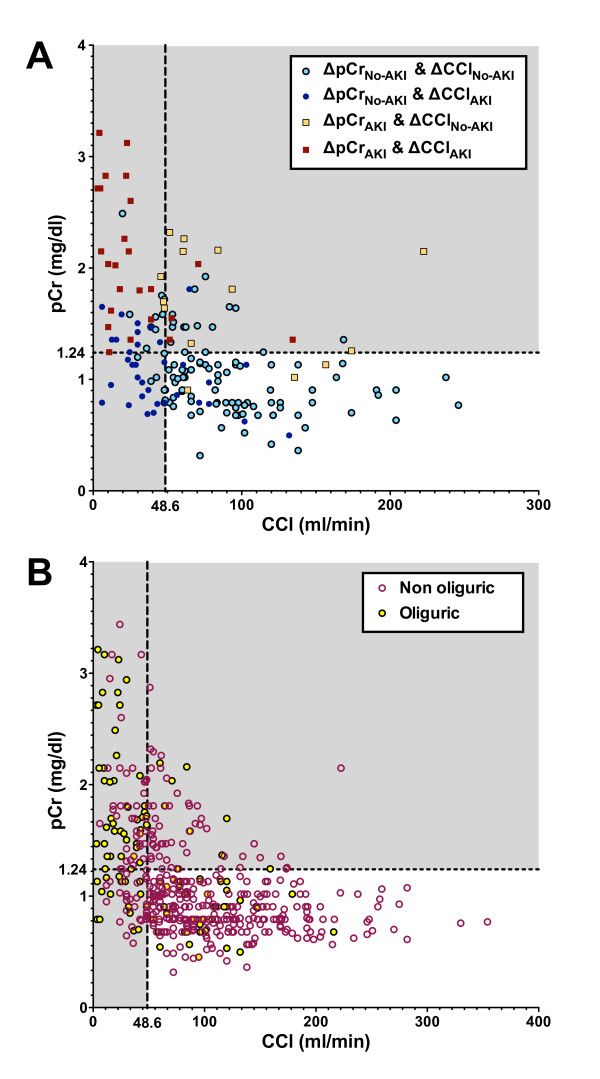
**Comparison of creatinine clearance (CCl) with plasma creatinine (pCr) on entry into the ICU in the (A) known baseline cohort and (B) entire cohort**. Dashed lines represent optimum cut-points for diagnosis of acute kidney injury (AKI) according to a change in CCl (CCl < 48.6 ml/min) or change in pCr (pCr > 1.24 mg/dl). **(A) **The four possible diagnoses (i) No AKI (ΔpCr_No-AKI _and ΔCCl_No-AKI_), (ii) AKI by the clearance criterion only (ΔpCr_No-AKI _and ΔCCl_AKI_), (iii) AKI by the pCr criterion only (ΔpCr_AKI _and ΔCCl_No-AKI_) or (iv) AKI by both criteria (ΔpCr_AKI _and ΔCCl_AKI_) are illustrated by squares for ΔpCr_AKI _and closed circles or squares for ΔCCl_AKI_. **(B) **Oliguric (urine output < 0.5 ml/kg/h average over 4 h, closed circles), and non-oliguric (open circles) for the entire cohort. No-AKI (CCl_No-AKI_): without AKI; ΔpCr: relative change in pCr from baseline; ΔCCl: relative change in CCl from Cockcroft-Gault (CG) baseline; AKI (pCr_AKI_): with AKI.

The optimal cut-point for CCl to diagnose ΔCCl_AKI _was 48.6 ml/min (AUC, 0.87; 95% CI, 0.81 to 0.94) calculated using 1/CCl) in the known baseline creatinine cohort (Figure 3). The optimal cut-point for pCr to diagnose ΔpCr_AKI _was 1.24 mg/dl (AUC, 0.91; 95% CI, 0.85 to 0.98). Below this cut-point adding CCl to a risk prediction model comprising pCr, UO and acute physiology and chronic health evaluation (APACHE) II scores (all *P *< 0.1 in a univariate analysis) for AKI (ΔCCl_AKI _or ΔpCr_AKI_) considerably improved the model: in the known baseline creatinine cohort the AUC increased by 0.23 to a moderate 0.77; a net 23% of those with AKI had greater risk whilst 60% of those without AKI had less risk, resulting in an NRI of 83%; the average increase in risk of those with AKI was 0.22 (IDI_AKI_) and the average decrease in risk of those without AKI was 0.074 (IDI_No-AKI_) indicating the model worked best to improve identification of those with AKI rather than exclude those without (Table 4). At a cut-point of CCl = 48.6 ml/min, the positive predictive value (PPV) was 0.88 and the negative predictive value (NPV) was 0.65.

**Table 4:** Risk reclassification using creatinine clearance plus clinical predictors (plasma creatinine, urine output, and APACHE II) compared with the clinical model alone for AKI on entry.

	**Known baseline creatinine cohort, pCr ≤ 1.24 mg/dl (*n *= 111**)
Comparison of model performance	

IDI_AKI_	0.22 (0.09 to 0.38)
IDI_No-AKI_	0.074 (0.029 to 0.13)
IDI	0.29 (0.12 to 0.49)
	
NRI_AKI_	23 (-9.2 to 56)
NRI_No-AKI_	60 (34 to 76)
NRI	83 (29 to 125)
	
Increase in AUC	0.23 (0.015 to 0.41)

Combined CCl and clinical predictors model performance	

AUC	0.77 (0.66 to 0.88)
IS	0.45 (0.29 to 0.61)
IP	0.16 (0.099 to 0.25)
PPV at < 48.6 ml/min	0.88 (0.81 to 0.94)
NPV at < 48.6 ml/min	0.65 (0.46 to 0.85)
Cut-point (ml/min) for PPV > 90%	< 78
Cut-point (ml/min) for NPV > 90%	< 38.7

Values in brackets represent 95% confidence intervals. APACHE: acute physiology and chronic health evaluation; IDI: integrated discrimination improvement; NRI: net reclassification improvement (continuous/category free); AUC: Area under the receiver operator characteristic curve (ideally = 1); Increase in AUC: difference in AUC between the combined model and the clinical predictors only model; CCl: creatinine clearance; IS: integrated sensitivity (ideally = 1); IP: integrated 1-specificity (ideally = 0); PPV: positive predictive value; NPV: negative predictive value.

### Prognosis on entry to ICU (entire cohort)

**Figure 4:**
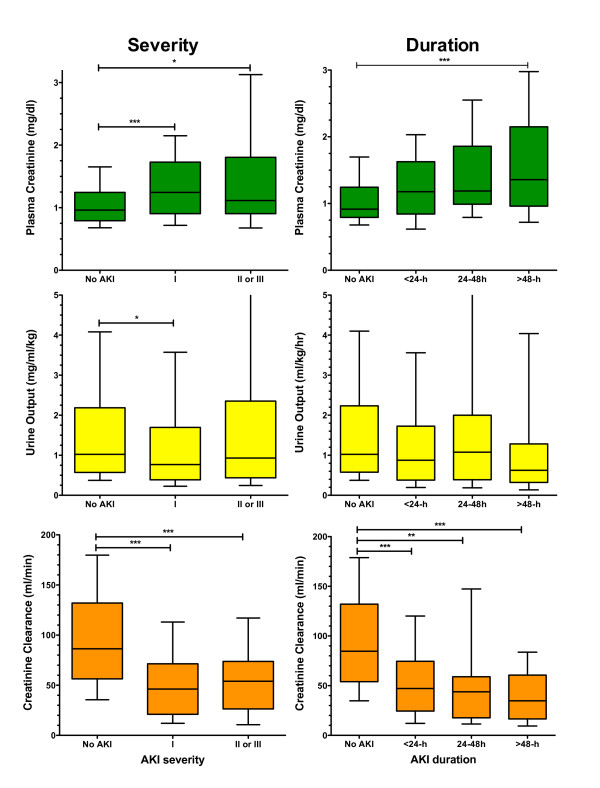
**Comparison of on entry plasma creatinine (pCr), urine output (UO), and creatinine clearance (CCl) in the entire cohort with: (i) AKI_AKIN _severity classes (No AKI, I, II or III) determined from on entry pCr and CCl and UO, (ii) Duration of AKI_AKIN_**. Post-hoc *** *P *< 0.001, ** *P *< 0.01, * *P *< 0.05.

On-entry CCl, pCr, and UO were associated with maximum severity of AKI observed over the next 48 h (AKI_AKIN_: *P *< 0.0001, *P *< 0.0001, *P *= 0.035 respectively, Mann-Whitney *U*- test) (Figure 4). CCl and pCr, but not UO were also associated with duration of AKI_AKIN _(*P *< 0.0001, *P *< 0.0001, *P *= 0.79 respectively).

On entry to the ICU, CCl moderately predicted the need for dialysis marginally better (AUC, 0.75; 95% CI, 0.59 to 0.91; *P *= 0.018) than pCr (AUC, 0.72; 95% CI, 0.56 to 0.89); UO was not predictive (AUC, 0.50; 95% CI, 0.41 to 0.75). CCl was predictive of death within 30 days, but with a lower AUC value of 0.61 (95% CI, 0.54 to 0.68). Neither pCr (AUC, 0.55; 95% CI, 0.48 to 0.62) nor UO (AUC, 0.55; 95% CI, 0.47 to 0.62) were predictive of death.

### Change in CCl after entry to ICU

CCl decreased by > 33.3% over the first 12 hours in 72 patients (14.9%) following ICU entry. These patients were more likely to require dialysis or die within 30 days than patients with smaller decreases (29% vs 15%, *P *= 0.0057; RR, 2.0; 95% CI, 1.3 to 3.0). Only sixteen patients (22%) had a subsequent increase in pCr of more than 50% as would be expected by creatinine kinetic modelling. The median (IQR) time for this increase from the on-entry sample was 25 (17 to 44) h. This was greater than the 16 h required to measure the 33.3% decrease in 4-h CCl required to diagnose AKI; *P *= 0.0046, Mann Whitney *U*-test. Of the other 56 patients, 21 had very high on-entry CCl (> 140 ml/min), two-thirds of whom were admitted following head injury, neurological surgery, stroke or Guillian Barre syndrome; 22 had a transient decrease in CCl (resolved by 24 h) which may explain the lack of increase in pCr; 3 exhibited elevations in pCr of 29 to 47% (that is, less than the 50% required for AKI classification); 3 patients did not exhibit any significant changes in pCr and seven had no subsequent CCl measurements.

Thirty-six patients exhibited a rise in pCr > 50% but no decrease in CCl over 12 h. Of these, 27 had a decrease in CCl on entry (23) or by 24 h (4) from the CG baseline greater than 33.3% (determined retrospectively); 5 had decreases of CCl > 33.3% between later time points (between day 2 and day 3) followed by an increase in pCr, and 4 showed no decrease in CCl preceding a increase in pCr.

## Discussion

The measurement of a brief CCl, relative to a known baseline value (or when unknown, relative to a calculated CG baseline value), provided earlier diagnostic and prognostic information, compared with change in pCr alone. AKI on entry to the ICU was identified in one third of patients not identified by pCr. After admission to the ICU, these patients were identified earlier than those identified by ΔpCr, consistent with the delay in pCr equilibration suggested by creatinine kinetics. ΔCCl also identified a cohort of patients, the (ΔpCr_AKI _and ΔCCl_No-AKI_) group, with normal renal clearance but with an increased pCr, consistent with recovering renal function after an earlier loss of GFR. This mistiming between CCl measurement and steady state pCr potentially explains the poor correlation (r^2 ^= 0.18) observed. The probability of death or dialysis was more closely associated with the ΔCCl RIFLE severity grade than with ΔpCr severity grade. Serial measurements of CCl provided a diagnosis of AKI and of improving renal function earlier than serial measurements of pCr and also identified patients at risk of dialysis.

A low on-entry CCl was associated with poor outcome, including death. In contrast, neither UO nor pCr predicted death. The moderate AUCs indicate that CCl is not a useful stand-alone predictor of death. When added to a risk prediction model for AKI in patients with low on-entry pCr (< 1.24 mg/dl), CCl greatly improved the model. A sub-threshold CCl predicted the development of AKI and was associated with severity of AKI when AKI was determined using the on-entry creatinine as baseline. The combination of a normal pCr (< 1.24 mg/dl) and low CCl (< 48.6 ml/min) had a moderately high predictive value (0.88) for the early detection of AKI.

Patients with a low CCl and increased pCr need additional information to distinguish on-entry AKI from chronic kidney disease (CKD) in the absence of a pre-admission baseline creatinine. The presence or absence of oliguria alone in this group was not sufficient to distinguish patients with CKD from those with AKI (data not shown). Underlying CKD causes the relative increase in pCr to be lower, potentially delaying diagnosis of AKI if a relative change in creatinine is used for diagnosis [24]. An absolute rise in creatinine allows diagnosis to be independent of the underlying function [24]. However, for early diagnosis, injury biomarkers not associated with CKD are needed to identify on-entry AKI from CKD without AKI. Unfortunately, amongst the injury markers we measured in this cohort (with time course of sufficient duration to be increased when pCr was increased) biomarker performance depended on baseline renal function [10] which makes interpretation difficult when the patient has CKD. Only 22% of patients (*n *= 16) with an initial one third decrease in CCl subsequently developed a 50% increase in pCr as predicted by the creatinine kinetic model. Serial measurements were needed to identify patients with only a transient decrease in CCl. Some of the false positives in this group reflected a high initial CCl, suggesting caution when interpreting a decrease in CCl if the initial CCl baseline is high (> 140 ml/min).

The optimum frequency for monitoring loss of GFR remains to be determined, and near real-time measurements of GFR are possible [8]. In this study, CCl was measured on entry to the ICU and then 12 hourly. Since urine output is measured hourly in most ICUs, the frequency of CCl measurements could easily be increased with more frequent plasma and urine creatinine sampling. CCl would then have the potential to provide earlier information on worsening of AKI severity and to facilitate interpretation of changes in pCr. For example, while a modest increase in pCr suggests progression of severity, for example, from RIFLE stage R to stage I, renal function may remain unchanged. An unchanged CCl would demonstrate that the apparent progression represented a prior, rather than a continuing decrease in GFR. Detection of a true decline in renal function requires demonstration of incremental loss of GFR, which may be detected by serial CCl measurements but not initially by serial pCr measurement. CCl may also be preferable to pCr for other clinical purposes, such as calculation of dose for renal-excretion drugs [25], or for triaging patients in trials in AKI, aiming to intervene after a decrease in GFR but prior to creatinine increase, so-called secondary prevention [26].

Alone, the urine output criterion for diagnosis of AKI was not a reliable alternative to CCl. Consistent with these findings, Prowle *et al*. recently demonstrated that most episodes (94%) of oliguria were not associated with AKI (RIFLE I by the creatinine- change criterion) the next day [27]. As discussed, there are many modifiers of urine output in critically ill patients, including the administration of fluids and diuretics, and impaired ability for urinary concentration in some patients with CKD.

The search for new biomarkers of AKI has aimed to identify injury that leads to significant loss of GFR, as an alternative to waiting for GFR-induced change in pCr. Nevertheless, most biomarker studies have relied on change in creatinine, the surrogate for change in GFR, on the assumption that pCr changes follow a simple creatinine kinetic model [28]. This was the assumption behind the RIFLE definition of equating a percentage increase in pCr with a percentage decrease in GFR [6,18]. Although the AKIN removed change in GFR from the RIFLE, we have argued that the principal of measuring a change in GFR should be retained as the gold standard in the definition of AKI [29]. While awaiting a commercially available real-time measure of GFR, we postulate that serial CCl may allow identification of the specific phase of injury in AKI [26]. Brief CCl could also help determine whether a particular injury biomarker was increased before or after a decrease in GFR, which may also facilitate appropriate intervention. In a recent AKI biomarker study, the difference between the estimated baseline and 12-h CCl within 48 h of ICU admission was used to define patients with AKI [30]. This enabled the study to assess the effectiveness of a combination of pCr and urinary gamma-glutamyltranspeptidase in AKI detection. This suggests that change in CCl may be independently useful as a selection criterion for early intervention.

A low CCl (in our study < 48.6 ml/min) on entry to the ICU indicates that individuals are at high risk of AKI. We suggest this should lead to the appropriate management recommended by the Kidney Disease: Improving Global Outcomes (KDIGO) consortium (see Figure 4 in [31]). This comprises: discontinuation of nephrotoxic agents, maintenance of perfusion pressure and volume status, further haemodynamic monitoring, continued monitoring of serum creatinine and urine output, avoidance of hyperglycaemia and contrast procedures, and additional diagnostic workups. In addition, we recommend further CCl monitoring. As a research tool, where available, measurement of biomarkers of kidney injury could assist in establishing the diagnosis of AKI [32,33]. In contrast, if CCl on entry to the ICU is normal in the presence of an increased pCr, patients may be stratified to lower risk. At this stage, we would recommend at least one more 4-h CCl to confirm normal clearance. Clearly, biomarkers of acute kidney injury could be helpful here too, if available. Clearly all patients who appear to have had a short episode of AKI prior to entry to ICU should have nephrology follow up after discharge to check for progression to CKD.

### Limitations

There are limitations in the use of a CCl to estimate GFR. A measured CCl is averaged over the collection period and requires pre- and post or mid-point pCr measurement. If the time interval is brief pCr is unlikely to change substantially. If the interval is too brief, relative errors in measurement of urine output may be increased. We suggest that 2 to 4 h is a reasonable and practical compromise. We recently demonstrated in these same patients that a 4-h clearance could detect the early phase decrease in CCl that characterises the initial phase of patients developing AKI [34]. The costs associated with CCl, are minimal, simply more frequent assays of urine and pCr, with careful attention to recording urine output accurately.

The optimal frequency for CCl measurements is uncertain. In a patient with oliguria, urine collection for less than 1 to 2 h will be relatively inaccurate. Assuming laboratory turnaround was 2 h or less, the maximum frequency of clearance measurements would be 3- to 4-hourly, which may set a reasonable minimum interval over which to review change based on creatinine excretion data. Clearly, given time and cost, these measurements should be undertaken on entry to the ICU to establish a baseline, but only repeated at this frequency in patients at high risk of, or already suspected of having AKI.

Few studies have compared short duration CCl in the ICU with direct measures of GFR. Robert *et al*. compared inulin clearance with 30-minute CCl in 20 consecutive ICU patients [35]. Whilst there was no statistically significant bias, the 95% CI of the difference was large (-88 to 74 ml/min) although halved by the removal of two apparent outliers. Wharton *et al*. compared inulin clearance, ^99 m^Tc-DTPA and 2-h CCl in 18 acute patients with AKI [36]. Again there was no bias. The 95% CI for the difference was from -33 to +16 ml/min for inulin and -20 to +11 ml/min for ^99 m^Tc-DTPA. Hilbrands *et al*. measured CCl during simultaneous inulin and EDTA clearance (1.5 h) [37]. CCl overestimated GFR by about 20%, a difference that disappeared following cimetidine administration. Hoste *et al*. compared 1-h CCl in ICU patients with normal pCr with CG and modified diet in renal disease (MDRD) estimations of GFR [11]. The difference between these equations and CCl was large and clinically significant. They concluded that these equations were not acceptable alternatives to measured CCl.

The rapid loss of GFR causes rapid changes in creatinine excretion [38]. Initially excretion falls in proportion to the loss of GFR followed by a gradual increase in excretion in proportion to the gradual increase in pCr concentration (see Figure five in [34]). CCl is therefore influenced initially by the fall in creatinine excretion, and later by any change in plasma concentration. Errors will be introduced by factors which independently alter creatinine excretion. For example, CCl overestimates GFR because of tubular secretion of creatinine, which approximates 10 to 20% at normal GFR but increases with declining GFR [37,39]. While cimetidine inhibition of tubular creatinine secretion improves GFR estimates [37,40], this may interfere with other drug excretion and is probably not useful in the timeframe of AKI. A change in renal blood flow which induces AKI may also directly modify creatinine secretion, but there is little evidence from which to quantify this. CCl is likely to be unreliable when there is polyuria, such as in diabetes insipidus or, when acute brain injury is causing cerebral salt wasting [41].

Finally, as with pCr, change in clearance requires a baseline value for interpretation. This will be absent in many cases. Estimation equations perform poorly when used to estimate a baseline creatinine [42-44]. Similarly, it will not always be possible to decide if a measured CCl on admission to the ICU reflects baseline renal function or a loss of function. We used the CG formula to estimate baseline clearance. Whilst other formulas are more precise estimates of GFR, it was appropriate to use an estimate of CCl as a baseline for calculation of change in CCl, potentially minimising some of the error introduced by creatinine secretion. Using the MDRD formula made minimal difference (results not shown). Additional clinical information is also required to separate those with CKD from those with AKI on entry to the ICU.

## Conclusions

In a high-risk clinical setting, short duration CCl measurement is useful for patients with a known baseline creatinine for whom a CG estimation of baseline CCl can be made or when pCr is low. In this setting, low CCl suggests an acute loss of renal function and will influence drug dosing, initiate avoidance of known nephrotoxins, and trigger early nephrology consultation. Regular additional CCl would monitor recovery. Further, larger studies are required to determine the optimum frequency and duration of CCl measurement.

When pCr is increased in a patient with normal baseline values, 4-hourly CCl can distinguish resolving and ongoing renal impairment.

CCl may be useful in clinical trials by identifying patients soon after loss of renal function and before pCr is elevated, or by excluding patients when renal function is impaired.

## Key Messages

• Repeated 4-h CCl in the ICU are viable

• Low 4-h CCl in the presence of a normal pCr indicates recent loss of renal function

• Normal 4-h CCl in the presence of an increased pCr indicates renal recovery

## Abbreviations

AKI: acute kidney injury; AKI (eg pCr_AKI_): with AKI; No-AKI (eg CCl_No-AKI_): without AKI; AKIN: Acute Kidney Injury Network; ANOVA: one-way analysis of variance; APACHE: acute physiology and chronic health evaluation; AUC: area under the receiver operator characteristic curve; CCl: creatinine clearance; ΔCCl: relative change in CCl from CG baseline; CG: Cockcroft-Gault; CKD: chronic kidney disease; EARLYARF: Early intervention in Acute Renal Failure trial; GFR: glomerular filtration rate; IDI: integrated discrimination improvement; IQR: interquartile range; KDIGO: Kidney Disease: Improving Global Outcomes; MDRD: modified diet in renal disease; pCr: plasma creatinine; ΔpCr: relative change in pCr from baseline; RIFLE: Risk, Injury, Failure, Loss, End stage; NPV: negative predictive value; NRI: net reclassification improvement; PPV: positive predictive value; RR: relative risk; UO: urine output.

## Competing interests

The authors declare that they have no competing interests.

## Authors' contributions

JP: analysis design, data analysis and manuscript drafting. CF: statistical design and manuscript approval. RW: EARLYARF trial design, data collection, and manuscript approval. GS: data collection, analysis design and manuscript approval. ZH: EARLYARF trial design, Principal Investigator, analysis design and manuscript drafting. All authors have approved the manuscript.

## References

[B1] Hoste EAJ Clermont G Kersten A Venkataraman R Angus DC de Bacquer D Kellum JA RIFLE criteria for acute kidney injury are associated with hospital mortality in critically ill patients: a cohort analysis Crit Care 2006 10 R73 10.1186/cc4915 16696865 PMC1550961

[B2] Cruz DN Bolgan I Perazella MA Bonello M de Cal M Corradi V Polanco N Ocampo C Nalesso F Piccinni P Ronco C North East Italian Prospective Hospital Renal Outcome Survey on Acute Kidney Injury NEiPHROS-AKI Investigators North East Italian Prospective Hospital Renal Outcome Survey on Acute Kidney Injury (NEiPHROS-AKI): targeting the problem with the RIFLE Criteria Clin J Am Soc Nephro 2007 2 418 425 10.2215/CJN.03361006 17699446

[B3] Ostermann M Chang RWS Acute kidney injury in the intensive care unit according to RIFLE Crit Care Med 2007 35 1837 43 quiz 1852 10.1097/01.CCM.0000277041.13090.0A 17581483

[B4] Ricci Z Cruz D Ronco C The RIFLE criteria and mortality in acute kidney injury: A systematic review Kidney Int 2008 73 538 546 10.1038/sj.ki.5002743 18160961

[B5] Bagshaw SM George C Dinu I Bellomo R A multi-centre evaluation of the RIFLE criteria for early acute kidney injury in critically ill patients Nephrol Dial Transpl 2008 23 1203 1210 10.1093/ndt/gfm744 17962378

[B6] Bellomo R Ronco C Kellum JA Mehta RL Palevsky PM Acute Dialysis Quality Initiative workgroup Acute renal failure - definition, outcome measures, animal models, fluid therapy and information technology needs: the Second International Consensus Conference of the Acute Dialysis Quality Initiative (ADQI) Group Crit Care 2004 8 R204 12 10.1186/cc2872 15312219 PMC522841

[B7] Mehta RL Kellum JA Shah SV Molitoris BA Ronco C Warnock DG Levin A Acute Kidney Injury Network Acute Kidney Injury Network: report of an initiative to improve outcomes in acute kidney injury Crit Care 2007 11 R31 10.1186/cc5713 17331245 PMC2206446

[B8] Endre ZH Pickering JW Walker RJ Clearance and beyond: the complementary roles of GFR measurement and injury biomarkers in acute kidney injury (AKI) Am J Physiol-Renal 2011 301 F697 707 10.1152/ajprenal.00448.2010 21753074

[B9] Coca SG Yalavarthy R Concato J Parikh CR Biomarkers for the diagnosis and risk stratification of acute kidney injury: a systematic review Kidney Int 2008 73 1008 1016 10.1038/sj.ki.5002729 18094679

[B10] Endre ZH Pickering JW Walker RJ Devarajan P Edelstein CL Bonventre JV Frampton CM Bennett MR Ma Q Sabbisetti VS Vaidya VS Walcher AM Shaw GM Henderson SJ Nejat M Schollum JBW George PM Improved performance of urinary biomarkers of acute kidney injury in the critically ill by stratification for injury duration and baseline renal function Kidney Int 2011 79 1119 1130 10.1038/ki.2010.555 21307838 PMC3884688

[B11] Hoste E Damen J Vanholder R Lameire N Delanghe J van den Hauwe K Colardyn F Assessment of renal function in recently admitted critically ill patients with normal serum creatinine Nephrol Dial Transpl 2005 20 747 753 10.1093/ndt/gfh707 15701668

[B12] Herget-Rosenthal S Kribben A Pietruck F Ross B Philipp T Two by two hour creatinine clearance - repeatable and valid Clin Nephrol 1999 51 348 354 10404695

[B13] Cherry R Eachempati S Hydo L Barie P Accuracy of short-duration creatinine clearance determinations in predicting 24-hour creatinine clearance in critically ill and injured patients J Trauma 2002 53 267 271 10.1097/00005373-200208000-00013 12169932

[B14] Herrera-Gutierrez ME Seller-Perez G Banderas-Bravo E Munoz-Bono J Lebron-Gallardo M Fernandez-Ortega JF Replacement of 24-h creatinine clearance by 2-h creatinine clearance in intensive care unit patients: a single-center study Intens Care Med 2007 33 1900 1906 10.1007/s00134-007-0745-5 17609929

[B15] Endre ZH Walker RJ Pickering JW Shaw GM Frampton CM Henderson SJ Hutchison R Mehrtens JE Robinson JM Schollum JBW Westhuyzen J Celi LA McGinley RJ Campbell IJ George PM Early intervention with erythropoietin does not affect the outcome of acute kidney injury (the EARLYARF trial) Kidney Int 2010 77 1020 1030 10.1038/ki.2010.25 20164823

[B16] Australian New Zealand Clinical Trials Registry http://www.anzctr.org.au/

[B17] Cockcroft D Gault M Prediction of creatinine clearance from serum creatinine Nephron 1976 16 31 41 10.1159/000180580 1244564

[B18] Pickering JW Endre ZH GFR shot by RIFLE: errors in staging acute kidney injury Lancet 2009 373 1318 1319 10.1016/S0140-6736(09)60751-0 19376434

[B19] Pencina MJ D'Agostino RB Vasan RS Evaluating the added predictive ability of a new marker: From area under the ROC curve to reclassification and beyond Stat Med 2008 27 157 172 10.1002/sim.2929 17569110

[B20] Pencina MJ D'Agostino RB Steyerberg EW Extensions of net reclassification improvement calculations to measure usefulness of new biomarkers Stat Med 2011 30 11 21 10.1002/sim.4085 21204120 PMC3341973

[B21] Pickering JW Endre ZH New Metrics for Assessing Diagnostic Potential of Candidate Biomarkers Clin J Am Soc Nephro 2012 doi: 10.2215/CJN.09590911 10.2215/CJN.09590911 22679181

[B22] DeLong E DeLong D Clarke-Pearson D Comparing the areas under 2 or more correlated receiver operating characteristic curves - a nonparametric approach Biometrics 1988 44 837 845 10.2307/2531595 3203132

[B23] Pickering JW Frampton CM Endre ZH Evaluation of trial outcomes in acute kidney injury by creatinine modeling Clin J Am Soc Nephro 2009 4 1705 1715 10.2215/CJN.00820209 PMC2774954 19729431

[B24] Waikar SS Bonventre JV Creatinine Kinetics and the Definition of Acute Kidney Injury J Am Soc Nephrol 2009 20 672 679 10.1681/ASN.2008070669 19244578 PMC2653692

[B25] Kirkpatrick CM Duffull SB Begg EJ Pharmacokinetics of gentamicin in 957 patients with varying renal function dosed once daily Brit J Clin Pharmaco 1999 47 637 643 10.1046/j.1365-2125.1999.00938.x PMC2014263 10383541

[B26] Pickering JW Endre ZH Secondary prevention of acute kidney injury Curr Op Crit Care 2009 15 488 497 10.1097/MCC.0b013e328332f66f 19823082

[B27] Prowle JR Liu YL Licari E Bagshaw SM Egi M Haase M Haase-Fielitz A Kellum JA Cruz D Ronco C Tsutsui K Uchino S Bellomo R Oliguria as predictive biomarker of acute kidney injury in critically ill patients Crit Care 2011 15 R172 10.1186/cc10318 21771324 PMC3387614

[B28] Chiou WL Hsu FH Pharmacokinetics of creatinine in man and its implications in the monitoring of renal function and in dosage regimen modifications in patients with renal insufficiency J Clin Pharmacol 1975 15 427 434 1133219 10.1002/j.1552-4604.1975.tb02364.x

[B29] Pickering JW Endre ZH RIFLE and AKIN--maintain the momentum and the GFR! Crit Care 2009 13 416 10.1186/cc8019 19818161 PMC2784346

[B30] Blasco V Wiramus S Textoris J Antonini F Bechis C Albanèse J Martin C Leone M Monitoring of plasma creatinine and urinary γ-glutamyl transpeptidase improves detection of acute kidney injury by more than 20% Crit Care Med 2011 39 52 56 10.1097/CCM.0b013e3181fa431a 21178528

[B31] KDIGO Clinical Practice Guideline for Acute Kidney Injury Section 2: AKI Definition Kidney Int Suppl 2012 2 19 36 10.1038/kisup.2011.32 PMC4089595 25018918

[B32] Haase M Devarajan P Haase-Fielitz A Bellomo R Cruz DN Wagener G Krawczeski CD Koyner JL Murray P Zappitelli M Goldstein SL Makris K Ronco C Martensson J Martling C-R Venge P Siew E Ware LB Ikizler TA Mertens PR The outcome of neutrophil gelatinase-associated lipocalin-positive subclinical acute kidney injury a multicenter pooled analysis of prospective studies J Am Coll Cardiol 2011 57 1752 1761 10.1016/j.jacc.2010.11.051 21511111 PMC4866647

[B33] Nickolas TL Schmidt-Ott KM Canetta P Forster C Singer E Sise M Elger A Maarouf O Sola-Del Valle DA O'rourke M Sherman E Lee P Geara A Imus P Guddati A Polland A Rahman W Elitok S Malik N Giglio J El-Sayegh S Devarajan P Hebbar S Saggi SJ Hahn B Kettritz R Luft FC Barasch J Diagnostic and prognostic stratification in the emergency department using urinary biomarkers of nephron damage a multicenter prospective cohort study J Am Coll Cardiol 2012 59 246 255 10.1016/j.jacc.2011.10.854 22240130 PMC3487165

[B34] Ralib AM Pickering JW Shaw GM Devarajan P Edelstein CL Bonventre JV Endre ZH Test Characteristics of Urinary Biomarkers Depend on Quantitation Method in Acute Kidney Injury J Am Soc Nephrol 2012 23 322 333 10.1681/ASN.2011040325 22095948 PMC3269182

[B35] Robert S Zarowitz BJ Peterson EL Dumler F Predictability of creatinine clearance estimates in critically ill patients Crit Care Med 1993 21 1487 1495 10.1097/00003246-199310000-00016 8403957

[B36] Wharton WW Sondeen JL McBiles M Gradwohl SE Wade CE Ciceri DP Lehmann HG Stotler RE Henderson TR Whitaker WR Measurement of glomerular filtration rate in ICU patients using 99mTc-DTPA and inulin Kidney Int 1992 42 174 178 10.1038/ki.1992.275 1635347

[B37] Hilbrands LB Artz MA Wetzels JF Koene RA Cimetidine improves the reliability of creatinine as a marker of glomerular filtration Kidney Int 1991 40 1171 1176 10.1038/ki.1991.331 1762320

[B38] Waikar SS Sabbisetti VS Bonventre JV Normalization of urinary biomarkers to creatinine during changes in glomerular filtration rate Kidney Int 2010 78 486 494 10.1038/ki.2010.165 20555318 PMC3025699

[B39] KDOQI Clinical Practice Guidelines for Chronic Kidney Disease: Evaluation, Classification, and Stratification http://www.kidney.org/professionals/kdoqi/guidelines_ckd/p5_lab_g4.htm 11904577

[B40] Hellerstein S Berenbom M Alon U Warady B Creatinine clearance following cimetidine for estimation of glomerular filtration rate Pediatr Nephrol 1998 12 49 54 10.1007/s004670050402 9502568

[B41] Tisdall M Crocker M Watkiss J Smith M Disturbances of sodium in critically ill adult neurologic patients: a clinical review J Neurosurg Anesthesiol 2006 18 57 63 10.1097/01.ana.0000191280.05170.0f 16369141 PMC1513666

[B42] Bagshaw SM Uchino S Cruz D Bellomo R Morimatsu H Morgera S Schetz M Tan I Bouman C Macedo E Gibney N Tolwani A Oudemans-van Straaten HM Ronco C Kellum JA Beginning and Ending Supportive Therapy for the Kidney (BEST Kidney) Investigators A comparison of observed versus estimated baseline creatinine for determination of RIFLE class in patients with acute kidney injury Nephrol Dial Transpl 2009 24 2739 2744 10.1093/ndt/gfp159 19349297

[B43] Siew ED Matheny ME Ikizler TA Lewis JB Miller RA Waitman LR Go AS Parikh CR Peterson JF Commonly used surrogates for baseline renal function affect the classification and prognosis of acute kidney injury Kidney Int 2010 77 536 542 10.1038/ki.2009.479 20042998 PMC2929703

[B44] Pickering JW Endre ZH Back-calculating baseline creatinine with MDRD misclassifies acute kidney injury in the intensive care unit Clin J Am Soc Nephro 2010 5 1165 1173 10.2215/CJN.08531109 PMC2893073 20498242

